# Design and Implementation of a Coastal-Mounted Sensor for Oil Film Detection on Seawater

**DOI:** 10.3390/s18010070

**Published:** 2017-12-28

**Authors:** Yongchao Hou, Ying Li, Bingxin Liu, Yu Liu, Tong Wang

**Affiliations:** 1 Navigation College, Dalian Maritime University, Dalian 116026, China; houyongchao@dlmu.edu.cn (Y.H.); gisbingxin@dlmu.edu.cn (B.L.); dlmuwtxxglyxxxt@163.com (T.W.); 2 Environmental Information Institute, Dalian Maritime University, Dalian 116026, China; liuyudmu@dlmu.edu.cn

**Keywords:** oil spills, ultraviolet fluorescence spectroscopy, relative fluorescence intensity, monitoring system

## Abstract

The routine surveillance of oil spills in major ports is important. However, existing techniques and sensors are unable to trace oil and micron-thin oil films on the surface of seawater. Therefore, we designed and studied a coastal-mounted sensor, using ultraviolet-induced fluorescence and fluorescence-filter systems (FFSs), to monitor oil spills and overcome the disadvantages of traditional surveillance systems. Using seawater from the port of Lingshui (Yellow Sea, China) and six oil samples of different types, we found that diesel oil’s relative fluorescence intensity (RFI) was significantly higher than those of heavy fuel and crude oils in the 180–300 nm range—in the 300–400 nm range, the RFI value of diesel is far lower. The heavy fuel and crude oils exhibited an opposite trend in their fluorescence spectra. A photomultiplier tube, employed as the fluorescence detection unit, efficiently monitored different oils on seawater in field experiments. On-site tests indicated that this sensor system could be used as a coastal-mounted early-warning detection system for oil spills.

## 1. Introduction

Marine environmental problems have attracted increasing attention in recent decades [1,2]. Petroleum products play an important role in modern society as a source of energy and chemical feedstock, as a result, oil spills inevitably occur during the production, use, transport and storage of petroleum products. One of the most complete studies of the spread and impact of oil spills on human activity, environmentally sensitive shorelines and offshore regions is the research of Alves et al. on the Mediterranean Basin [3,4,5]. In particular, oil production platforms and oil storage tanks in coastal areas are major sources of oil spills, which can affect various aspects of daily human activities [6,7]. Therefore, routine surveillance in major ports is important for the early warning, prevention and control of oil spills. There are various modelling techniques applied to simulate the behavior of oil spills. Alves et al. modelled the behavior of oil from spills located at various depths below the sea surface [8,9]. In addition, excellent laboratory and remote sensing techniques exist for the remote detection of oil spills [10,11,12,13,14]. For such applications, techniques and sensors are required that permit high-frequency monitoring in the field, at a low cost per analysis. For example, Chase et al. have developed an oil spill detection and alarm system that can detect trace oil and micron-thin oil film on the surface of seawater, in real time [15]. It is difficult to achieve stable and low-cost measurements when continuous monitoring is desired. Further, producing sensor with anti-corrosion properties and a low explosive risk is challenging, given the hazardous nature of old oil storage tanks.

Optical measurement techniques are well suited to this purpose, since they permit continuous surveillance [16]. Excitation-emission matrix spectroscopy (EEMS) is an effective tool for the detection and quantification of oils on the surface of seawater [17]. In EEMS, ultraviolet (UV) lasers are used as the excitation source in the spectral region between 240 and 355 nm and emission spectra are collected in the wavelength region between 300 and 500 nm. EEMS require large, specialized laboratory equipment to acquire the fluorescence spectra, which requires a dedicated power source. Thus, EEMS are not practical for many field applications. Among the various optical principles available for sensing, UV-induced fluorescence methods have an advantage in that almost all oil types have characteristic fluorescence properties and fluorescence can be used to obtain valuable information required to detect oil on various backgrounds, including seawater, soil, ice and snow [18,19]. For example, fluorescence spectra and light absorption properties have been obtained by UV-induced fluorescence methods to detect trace oil on seawater. These advantages have led to the development of a variety of laboratory and field instruments [20,21].

Fiber optical sensors can be used with UV lasers for the fluorescence detection of organic pollutants in water, especially oil products [22,23,24]. A multi-channel receiver, fluorescence LiDAR, or photomultiplier tube (PMT) can be used to detect and record the emitted fluorescence spectrum. When combined with fiber optics, in situ applications and long-distance surveillance systems are feasible [25]. However, for short wavelengths in the UV spectral range, laser transmission is limited by increased fiber attenuation in the fiber material. The transmission quality will decrease when intense UV light propagates through the commonly used fused silica fibers [26]. In addition, fiber optics need more maintenance for day and night surveillance. These factors set limitations on the long-distance detection and monitoring of oil spills.

In this study, we considered the UV-induced fluorescence of both clean and oil-bearing seawater to examine the efficacy of applying the coastal-mounted fluorescence filter system-based (FFS-based) method for the detection and monitoring of oil spills on the surface of seawater. Our goal was to develop a compact, sensor-based instrument with day-long operability that could be mounted at a coastal site such as a harbor or port. This sensor-based instrument complements ‘late’ spill modelling, once the oil spill has reached the shore or is very close to it. This paper describes the design of the coastal-mounted FFS instrument and the experiments to evaluate its performance.

## 2. Design and Implementation of FFS-Based Coastal-Mounted Sensor

**Figure 1:**
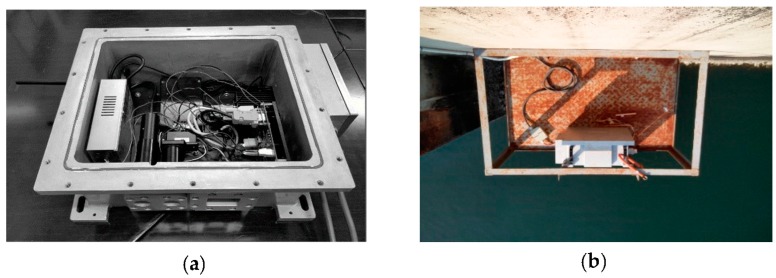

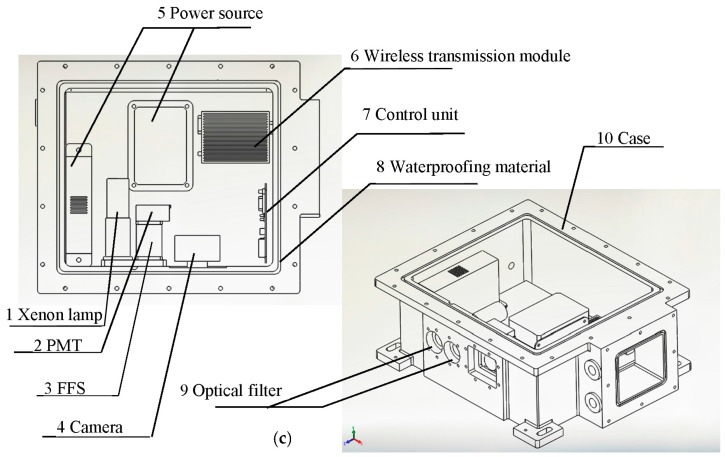
Schematic diagram of the coastal-mounted sensor for monitoring oil spills: (**a**) the internal structure; (**b**) installation drawing; and (**c**) structural sketch of the coastal-mounted sensor.

Figure 1 presents schematic diagrams of the coastal-mounted sensor for monitoring oil spills. The continuous wave source is a xenon lamp (200–300 nm, L4634-01 synthetic silica glass, Hamamatsu Photonics, Hamamatsu, Japan) with a dedicated power source (C13315, Hamamatsu Photonics). For this study, we chose a PMT (H10723-110, Hamamatsu Photonics) as the real-time detection unit. The sensor was also required to meet a number of constraints, including a compact size, low power consumption and independence from outside water and power requirements. These subassemblies are compact and integrated within a stainless-steel case (roughly 45 × 40 × 20 cm) which has anti-corrosion and anti-explosion properties. The coastal-mounted sensor can detect micron-thin oil films in the laboratory and can operate at range in excess of 5-m above the seawater surface.

The result of experiments in initial design and upgrade of sensor indicated that the xenon lamp and PMT can be proved to be highly effective for detection of micron-thin oil film (the minimum oil film thickness is 1.0 μm) from a distance of 5-m above the target surface area. The key limitation for detection range was that the excitation intensity of xenon lamp is whether enough to enable oil film to be detected by the PMT. Besides, this 5-m limit is the approximate upper bound for reliable detection. The coastal-mounted sensor can achieve reliable detection once per second (maximum frequency) determined by the sensor’s control unit. Wireless transmission module is required for the coastal-mounted sensor, which has been designed to use a basic RS232/RS485 protocol for integration with industrial process control systems. The main parameters of the coastal-mounted sensor are showing in Table 1.

**Figure 2:**
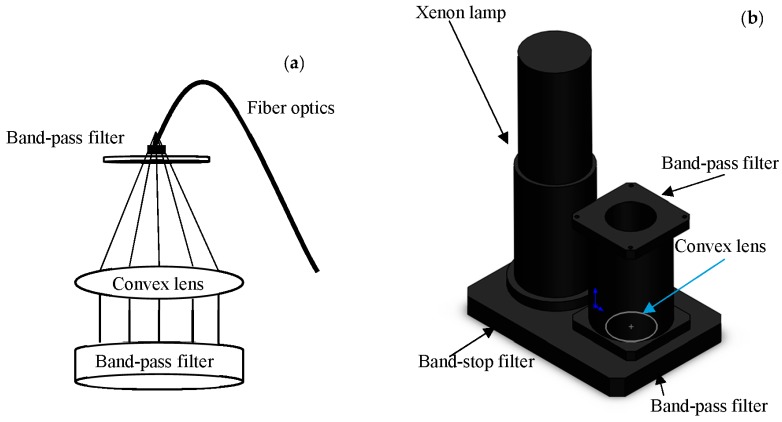
Schematic diagram of the FFS: (**a**) the optical schematic and (**b**) structural sketch of the FFS combined with the continuous wave source.

Previous studies on oil-on-seawater monitoring showed that fluorescence signals were affected by solar radiation and the choice of continuous wave source. These fluorescence signals were unable to detect trace oil and micron-thin oil films, unless the signal was filtered and processed [27,28]. In this study, a FFS was designed to improve the accuracy and reduce noise effects in the coastal-mounted sensor (Figure 1, No. 3). The FFS is composed of two-stage band-pass optical filters (300–400 nm) and a convex lens, as shown in Figure 2.

Selection of the 300–400 nm band-pass filter was made considering the impacts of solar radiation, as well as the fact that most oils have a fluorescence peak within this wavelength band. Further, the reflection coefficient of the ocean surface to the light in this band is less than that for the 400–600 nm band [29,30]. The first-stage optical filter is larger in size to expand the viewing angle. The convex lens is used to concentrate the fluorescence signals and thus converge on a single point in the center of the second optical filter. For detection, a spectrograph’s (laboratory) or PMT’s (field) fiber optics are connected to the center of the second optical filter (Figure 2a). To ensure the FFS receives fluorescence that is not affected by the continuous wave source, it is necessary to install a band-stop filter (300–400 nm) at the continuous wave source (Figure 2b).

## 3. Experiments

### 3.1. Seawater and Oil Samples

Seawater samples were collected in 80 mL glass bottles in September 2017 at the end of the largest berth in the port of Lingshui, China, on the northern Yellow Sea. Considering the possible presence of fluorescent substances (e.g., phytoplankton, algae) in clean seawater, enough seawater was collected to obtain background values.

Three main types of oils must be considered in studies of petroleum transport and storage: light fuel oil (diesel), heavy fuel oil and crude oil. We used −10# diesel oil, 0# diesel oil; 180# fuel oil, 380# fuel oil; Saudi crude oil and Brazilian crude oil, to represent these different oil types (Table 2).

**Figure 3:**
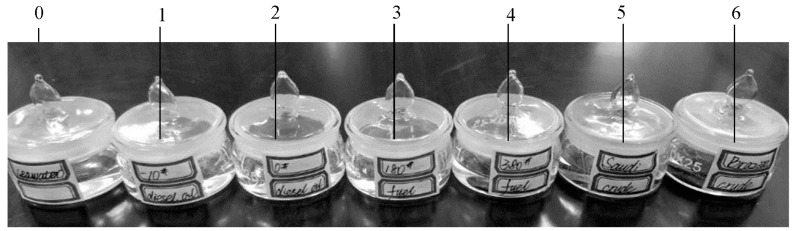
Laboratory samples used to simulate oil spills on seawater.

To simulate an oil spill on seawater, a small amount of each oil type was individually placed into six 40 mm × 25 mm glass bottles, along with 5 mL seawater, to form a 5 μm thick oil film (Figure 3). The amount of each oil was calculated using:

(1) $$V=\pi \cdot R^{2}\cdot h,$$

where R is the diameter of the vial (40 mm) and h is the desired computational thickness of the oil film (5 μm). These parameters afford the requisite oil volume of 6.28 μL. Bottles and lids with ground glass joints were selected to prevent oil volatilization. Additionally, a 40 mm × 25 mm glass bottle containing only seawater was used to determine the background fluorescence value for clean seawater.

### 3.2. Measurement and Apparatus

Both laboratory and field experiments were conducted. In the laboratory, the sensor’s FFS was connected to the fiber-optics of a 163-mm focal length Czerny-Turner spectrograph (Shamrock 163 Andor Technology, Northern Ireland, UK) and fluorescence was detected over a spectral range of 180–850 nm with a resolution of 5 nm (laboratory-mounted sensor). Ambient natural light was used as the environmental background in order to simulate the oil spill detection environment of the coastal areas in the laboratory. The detection distance was set at 1 m and all measurements were conducted under the same conditions.

For the field experiments, the sensor’ FFS was connected to a PMT (H10723-110, Hamamatsu Photonics) before installation in Lingshui Port, China (coastal-mounted sensor). This sensor for monitoring oil spills was installed on the bank of the largest berth at a height of 1 m from the highest water line. Then, the coastal-mounted sensor was employed in simulated oil spill experiment as well as a continuous operation trial. The monitoring data were stored and analyzed.

## 4. Results and Discussion

### 4.1. Fluorescence Spectra of Clean Seawater and Six Oil Samples

**Figure 4:**
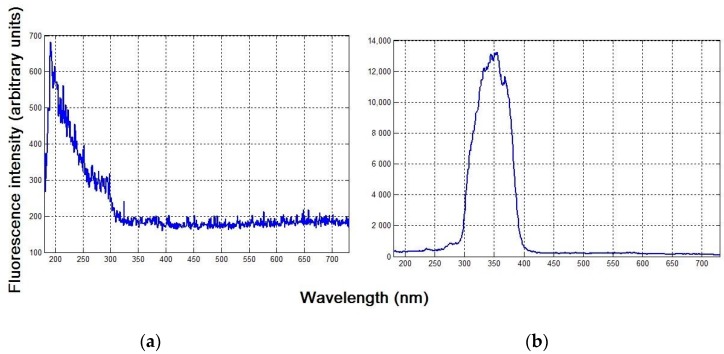
Fluorescence spectra of clean seawater: (**a**) without and (**b**) with the sensor’s FFS.

Figure 4 presents the fluorescence spectra of clean seawater after excitation in the absence and presence of the laboratory-mounted sensor’s FFS. As shown in Figure 4a, the spectrum is quite noisy and its intensity is low, preventing the retrieval of useful information. The fluorescence spectrum is also more strongly affected by the excitation source at wavelengths below 300 nm, where higher intensity is observed. In Figure 4b, the suppression of the influence of the ambient light and fluorescent signals outside the 300–400 nm band is clearly visible by the FFS of the laboratory-mounted sensor, showing enhanced fluorescence intensity in the desired region.

**Figure 5:**
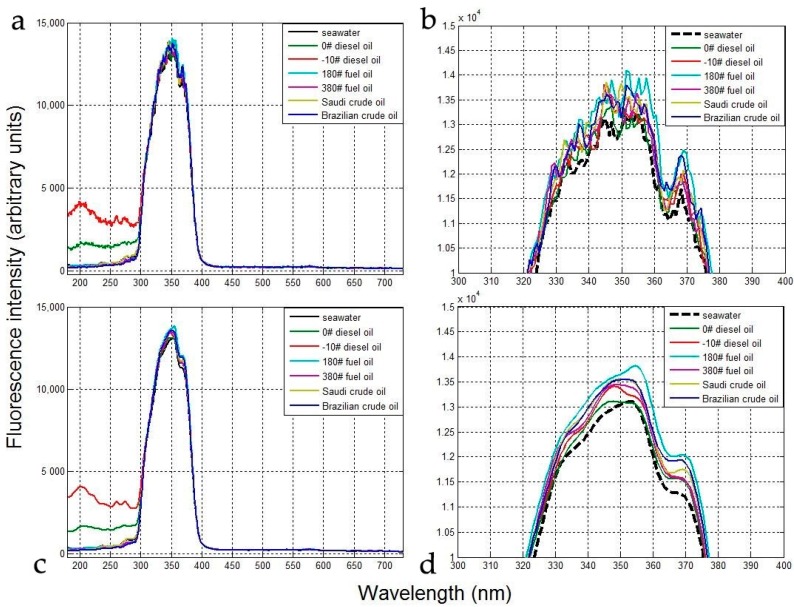
Fluorescence spectra of clean sea water and six oil samples determined with the laboratory-mounted sensor: (**a**) full data; (**b**) magnified area above fluorescence intensity of 10,000 and wavelength of 300–400 nm; (**c**,**d**) same as (**a**,**b**), respectively, but after data smoothing.

Figure 5a,b presents the fluorescence spectra of the clean seawater and six oil samples determined using the laboratory-mounted sensor and Figure 5c,d shows the same data after locally weighted smoothing (LOESS). The peaks detected in the figure correspond with EEMS spectra typical of seawater polluted by oils [31,32]. The fluorescence spectra of the clean sea water and six oil samples exhibit one main peak from 300–400 nm (centered at 350 nm). Thus, even small amounts of the oils produce intensity differences in the main peak of the fluorescence spectrum and all are distinct from clean sea water. All six samples and the blank exhibit another small peak at around 370 nm (Figure 5b,d), where the fluorescence intensity of the clean sea water is less than that of the oil samples. This peak is also sufficiently pronounced to differentiate among the different types of oil.

As seen in Figure 5, the fluorescence intensities for 0# diesel oil and −10# diesel oil are stronger than those from clean sea water and the other four oil samples in the 180–300 nm region, whereas the wavelength of UV-induced fluorescence for diesel oil is shorter than those of clean sea water and other oil types for a given fluorescence spectral wavelength. The detected fluorescence intensity of the diesel oils below 300 nm correlates with the fluorescence properties of mineral oil [31].

The fluorescence intensity decreases rapidly outside the wavelength range of the band-pass filter (300–400 nm) in the laboratory-mounted sensor, as the filter in the FFS suppresses the influence of both ambient light and fluorescence signals outside this wavelength band. However, the main peak determined for the six oil-on-seawater samples partially overlaps the clean sea water peak, making the detection of oil spills on seawater difficult. Therefore, it is necessary to determine an efficient method for accurately detecting oil spills on seawater.

In view of these results, it was necessary to find a parameter to enhance the difference between the fluorescence intensities of the oil samples and clean sea water in the main peak band (300–400 nm), so as to effectively detect various types of oil. To this end, we employed the relative fluorescence intensity (RFI), expressed as the difference between the fluorescence intensity of an oil-on-seawater sample and that of clean sea water:

(2) $$\mathrm{RFI}=I_{\left(intensity\;of\;oil\;on\;seawater\right)}-I_{\left(intensity\;of\;plain\;seawater\right).}$$

**Figure 6:**
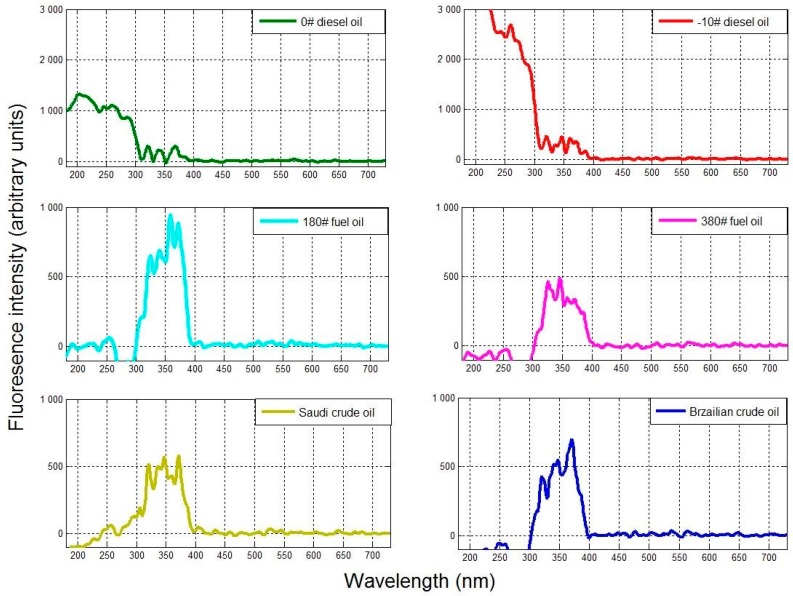
Relative fluorescence intensity (as defined by Equation (2)) for all six oil samples.

The RFIs for the six oil samples are shown in Figure 6. In each case, the main RFI peak (from 300–400 nm) is clearly visible. Moreover, the RFIs for the light diesel oils are significantly higher than those of the heavy fuel and crude oils in the 180–300 nm range (>1000, Figure 6), as opposed to the 300–400 nm range, in which the RFI values for the diesel oils are far lower (<500, Figure 6). The heavy fuel and crude oils exhibit the opposite trend.

Therefore, heavy fuel and crude oils can be effectively detected using the RFI in the 300–400 nm range, while light diesel oils can be detected in the 180–300 nm spectral range. Thus, the selection of a 300–400 nm band-pass filter and use of the RFI parameter can be effective for the detection of the different oil types’ fluorescence signals and the inhibition of background fluorescence.

### 4.2. FFS-Based Coastal-Mounted Sensor for Operation and Experiment

After demonstrating the potential of the lab-mounted system for oil detection on seawater, it was necessary to design an efficient FFS-based coastal-mounted sensor for routine oil-spill surveillance in major ports. One problem with detecting oil pollution in marine environments is the lack of sensitivity of in situ measurements. Figure 5 shows that the main peak determined for different oil types on seawater partially overlaps the peak for clean sea water. Therefore, a PMT was applied to determine the RFI in order to capture and enhance the spectral characteristics of oil spills on seawater and to take into account the fluorescence intensity of clean sea water.

**Figure 7:**
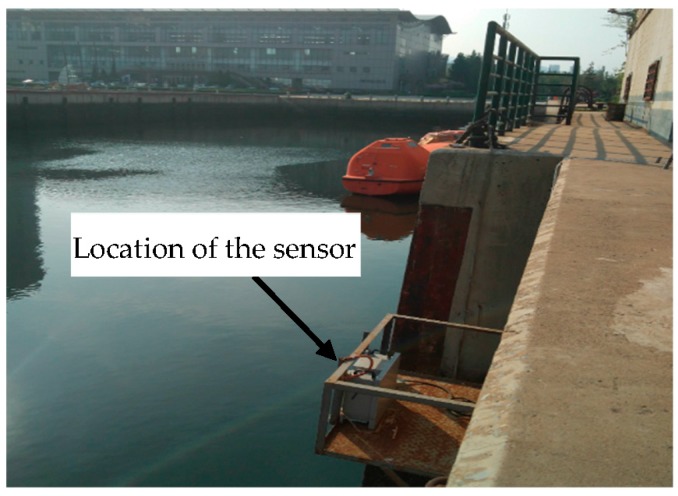
Location of the sensor in Lingshui port, China.

We investigated the feasibility of the designed instrument through continuous operation trials and routine application demonstrations in the port of Lingshui, China, from September–November 2017. The sensor was installed at the edge of the port’s largest berth, 1 m above the highest water line (Figure 7) and the monitoring data were stored and analyzed.

**Figure 8:**
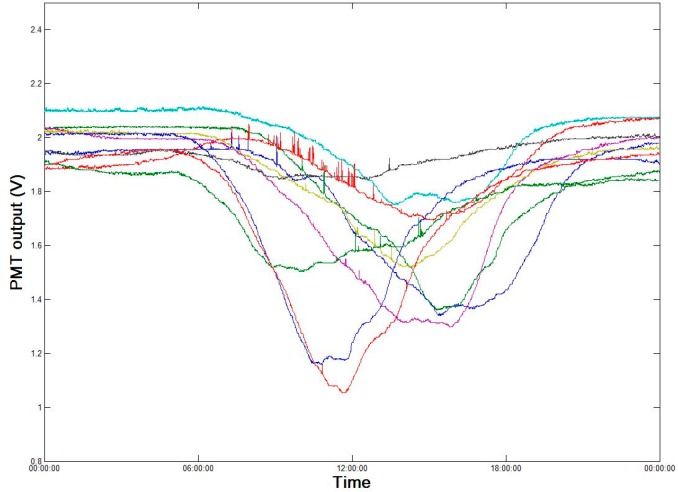
Daily monitoring data using PMT for ten days (each line represents a separate day).

Figure 8 presents the continuous daily monitoring data over a period of ten days (15–24 September 2017), during which the fluorescence intensities showed a clear daily reduction from 6:00 to 18:00. In other words, the received fluorescence intensity was lower during periods of strong daily solar radiation. This was due to the filtering of the background environmental fluorescence during sensor signal processing. The sensor control unit controls the PMT sampling frequency at a level greater than the continuous wave source flash frequency. The PMT first collects the fluorescence background value, $f_{0}$, of the environment and saves this value. After the excitation light source emits UV light, the original fluorescence signal of the detection area excited by the continuous wave source, $f_{initial}$, is collected and the actual fluorescence intensity (PMT output) is determined as $f_{actural}=f_{initial}-f_{0}$. Because solar radiation is strong during the day, the fluorescence background value of the environment is relatively high. When the environmental background level is removed from the actual monitoring value, it reduces the monitored fluorescence intensity. Consequently, false alarms can be avoided when environmental background values are high. Based on preliminary simulated oil spill experiments, the occurrence of oil spills can still be effectively monitored after removing the environmental fluorescence background values.

**Figure 9:**
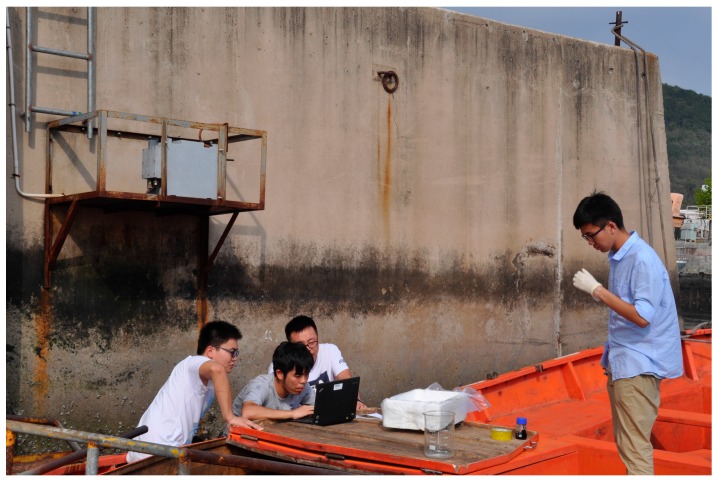
Observation site used for the simulated oil spill experiment.

**Figure 10:**
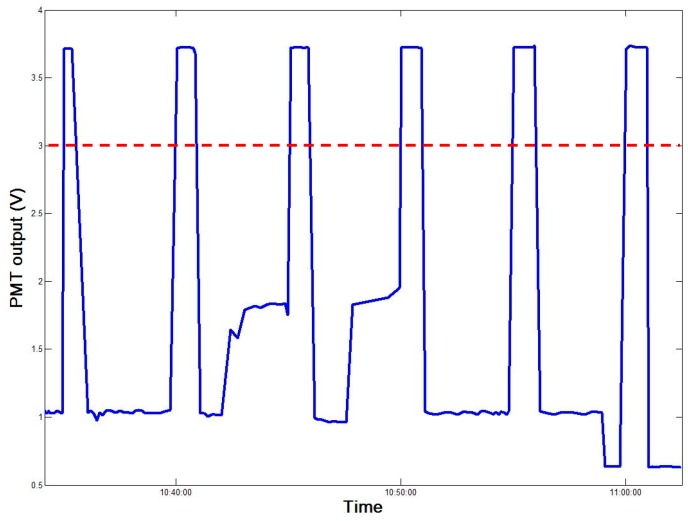
PMT recorded for the six oil types in sequential oil-spill simulations.

On 2 September 2017, simulated oil spill experiments were performed at the observation site (Figure 9). An equal quantity by volume of each of the six oil types was independently added to a bucket containing clean sea water from the harbor. The bucket was then placed in the detection area once each oil sample had formed a stable oil film and the monitoring data were recorded on a computer. After storage and display by computer software using wireless data-transmission technology, the PMT monitoring value was consistently above 3 V (Figure 10). When the FFS-based coastal-mounted sensor was turned on, oil placed in the detection area resulted in a rapid increase in fluorescence intensity. A strong fluorescence signal was detected by the PMT during the simulated oil spills. After repeated experiments, a PMT output value of 3 V was set in the sensor as the threshold for the occurrence of an oil spill. When this threshold is exceeded, the coastal-mounted sensor will issue an oil spill alarm.

**Figure 11:**
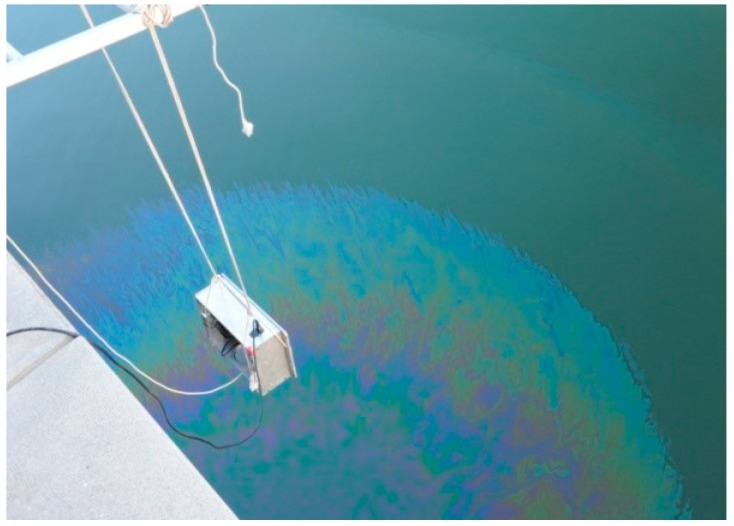
Observation site used for the continuous oil spill experiment.

**Figure 12:**
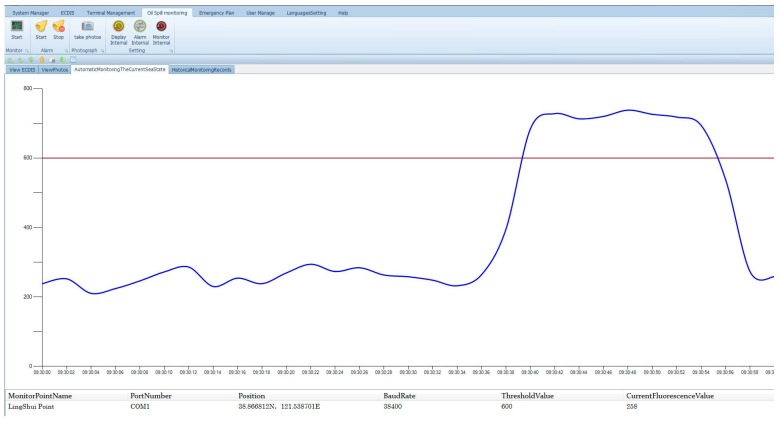
The monitor data in the continuous oil spill experiment.

Moreover, continuous oil spill experiments were performed at the observation site before the installation of sensor (Figure 11). A certain amount of 0# diesel (10 mL) is poured into the sea where oil placed in the sensor’s detection area. The monitoring data were recorded and displayed by computer software (Figure 12), which enables the sensor to effectively detect trace oil with the spread of spilled oil.

It should be stressed that the RFIs of the fluorescence spectral wavelength for the light diesel oils are significantly higher than those of the heavy fuel and crude oils in the 180–300 nm range on seawater, which was applied to differentiate the oils in the laboratory [33,34]. The PMT detection unit used in the sensor can effectively and accurately monitor low concentrations of oil [27,35]. Overall, this system is effective and can accurately monitor trace oils and micron-thin oil films on seawater and in other aquatic environments, thus overcoming the limitations of traditional monitoring technologies.

## 5. Conclusions

In this study, we used a continuous wavelength (200–300 nm) source with a xenon lamp and 300–400 nm band-pass filters to determine the UV-induced fluorescence spectra of clean sea water and six oil samples, representing three characteristic oil types. The RFIs were determined using the FFS-based coastal-mounted sensor and spectrograph from 180–300 nm (fluorescence maximum for diesel oils) and from 300–400 nm (fluorescence maximum for heavy fuel and crude oils). The RFI values for all oil types were substantially higher than for clean sea water, demonstrating that the FFS-based coastal-mounted sensor using the RFI can be effective for the daily routine monitoring of surface oil spills along coasts and in harbors, even when different types of oil are present. Furthermore, based on the FFS and PMT, the coastal-mounted sensor can effectively inhibit background light interference. Even in environments with strong solar radiation, the sensor accurately differentiated between oil spills and the clean sea water surfaces.

In addition to monitoring oil spills in harbor areas, this effective system could be used to continuously monitor reservoirs, drains, sewage tanks and other aquatic environments where oil products may leak, overcoming the limitations of traditional monitoring technologies.

## Figures and Tables

**Table 1:** The main parameters of the coastal-mounted sensor.

The detection subassemblies	Excitation wavelength	200–300 nm
	Detection wavelength	300–400 nm
	Detection range	0–5 m
	Detection frequency	0.1–1 Hz
	Temperature range	−20–+70 °C
The case of the sensor	Size	roughly 45 × 40 × 20 cm
	Material	stainless-steel
	Protection level	IP66

**Table 2:** Oil samples used in this paper.

Oil Type	Oil Sample	API	Number
Light fuel oil	−10# diesel oil	38.2	1
	0# diesel oil	38.2	2
Heavy fuel oil	180# fuel oil	11.3	3
	380# fuel oil	11.3	4
Crude oil	Saudi crude oil	33.7	5
	Brazilian crude oil	18.1	6
